# Genomics, genetics and breeding of common bean in Africa: A review of tropical legume project

**DOI:** 10.1111/pbr.12573

**Published:** 2018-04-17

**Authors:** Clare Mukankusi, Bodo Raatz, Stanley Nkalubo, Fenta Berhanu, Papias Binagwa, Michael Kilango, Magdalena Williams, Katungi Enid, Rowland Chirwa, Steve Beebe

**Affiliations:** ^1^ International Centre for Tropical Agriculture (CIAT) Kampala Uganda; ^2^ International Centre for Tropical Agriculture (CIAT) Cali Colombia; ^3^ National Crops Resources Research Institute (NaCRRI) Kampala Uganda; ^4^ Melkassa Agricultural Research Centre Oromia Region Adama town Ethiopia; ^5^ Selian Agricultural Research Institute (SARI) Arusha Tanzania; ^6^ Uyole Agricultural Research Institute (ARI‐Uyole) Mbeya Tanzania; ^7^ Maruku Agricultural Research Institute (ARI‐Maruku) Kagera Tanzania; ^8^ International Centre for Tropical Agriculture (CIAT) Lilongwe Malawi

**Keywords:** breeding tools, common bean, demand‐led, micronutrient content, production constraints

## Abstract

Common bean (*Phaseolus vulgaris* L.) is an important legume crop worldwide. The International Centre for Tropical Agriculture (CIAT) and its national partners in Africa aim to overcome production constraints of common bean and address the food, nutrition needs and market demands through development of multitrait bean varieties. Breeding is guided by principles of market‐driven approaches to develop client‐demanded varieties. Germplasm accessions from especially two sister species, *P. coccineus* and *P. acutifolius,* have been utilized as sources of resistance to major production constraints and interspecific lines deployed. Elucidation of plant mechanisms governing pest and disease resistance, abiotic stress tolerance and grain nutritional quality guides the selection methods used by the breeders. Molecular markers are used to select for resistance to key diseases and insect pests. Efforts have been made to utilize modern genomic tools to increase scale, efficiency, accuracy and speed of breeding. Through gender‐responsive participatory variety selection, market‐demanded varieties have been released in several African countries. These new bean varieties are a key component of sustainable food systems in the tropics.

## 1 INTRODUCTION

**Figure 1:**
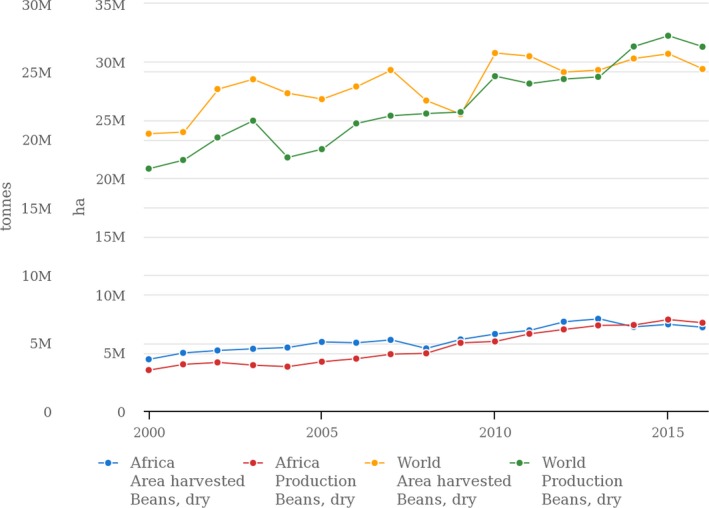
Common bean production (tonnes) vs. area under beans (ha) worldwide and in Africa over a 15 year period (2000‐2015) [Colour figure can be viewed at http://www.wileyonlinelibrary.com]

Common bean (*Phaseolus vulgaris* L.) is grown on about 30 million hectares globally and on 7.6 million ha in Africa annually where it is consumed and traded by more than 100 million households (Buruchara et al., 2011; FAOSTAT, 2014). Being a major staple, common bean contributes to health, food and nutritional security as it is well‐endowed with starch, protein, fibre and minerals such as iron, zinc, potassium, selenium, molybdenum and vitamins (thiamine, vitamin B6) and folate. It is an ideal crop for the smallholder farming systems due to its capability to fix N, short maturity period (≤3 months), easily converted to cash to meet urgent household needs, relatively long storage and convenience of handling the harvest and its compatibility with other crops (maize, cassava, banana, etc.), in many low‐input production systems. Three East African countries, Kenya, Tanzania and Uganda, are among the global leaders of common bean production (Akibode & Maredia, 2011; FAOSTAT, 2016). The per capita consumption of 40–60 kg/year in Rwanda, Kenya and Uganda is the highest in the world (Beebe, Rao, Blair, & Acosta‐Gallegos, 2013; Broughton et al., 2003). A unique partnership model involving CIAT and its research partners, together with effective breeding and seed delivery strategies, have helped to reach millions of beneficiaries with improved bean varieties (Buruchara et al., 2011). There is a notable increase in bean production in most African countries in the last 10 years most likely as a result of an increase in the area planted (Figure 1). However, on‐farm productivity remains low averaging of 850 kg/ha (Figure 1; FAOSTAT, 2016) compared to 2.5–5 t/ha that is achievable (Muthoni et al., 2017). More market‐driven African countries report higher productivity (yield/ha) probably because they are able to adopt and use improved crop technologies (Table 1) due to the assurance of market.

**Table 1:** Dry bean production in selected African countries

Area	Area harvested (ha)	Production (tonnes)	Yield (kg/ha)
Ethiopia	323,326	513,725	1588.9
South Africa	55,820	82,130	1471.3
Cameroon	266,543	362,055	1358.3
Uganda	674,000	876,576	1300.6
Ghana	165,720	201,150	1213.8
Madagascar	73,017	80,841	1107.2
United Republic of Tanzania	1,134,394	1,114,500	982.5
Rwanda	465,865	415,259	891.4
Burundi	380,592	251,761	661.5
Kenya	1,052,408	615,992	585.3
Malawi	329,959	188,745	572
Democratic Republic of the Congo	459,100	248,957	542.3
Mozambique	455,400	186,065	408.6
Zimbabwe	69,651	27,414	393.6

FAOSTAT database (Source: FAOSTAT, 2014).

## 2 ADDRESSING MAJOR CONSTRAINTS TO BEAN PRODUCTION

The low yield growth rates shown in Figure 1 could be attributed to a number of field‐based production constraints (Beebe et al., 2011). Common bean is typically not well adapted to extreme environments of heat, drought and excessive rainfall. Impacts of climate change on agriculture and bean productivity in particular have been discussed by Beebe et al. (2011), Boko et al. (2007), Christensen, Carter, Rummukainen, and Amanatidis (2007) and IFAD (2011) among other authorities. Crop improvement through breeding brings immense value relative to investment and offers an effective approach to improving food security (Tester & Langridge, 2010). Since 1996, CIAT's bean research and in particular the development of improved bean varieties for the smallholder farmers in SSA have been coordinated through the Pan Africa Bean Research Alliance (PABRA (http://www.pabra-africa.org) (Buruchara et al., 2011). Common bean breeding programmes in PABRA are hinged on three thematic areas: (i) improved dry bean varieties resistant to multiple environmental (biotic/ abiotic) and climate‐change related stresses; (ii) micronutrient‐rich bean varieties and (iii) high value bean varieties targeted to niche markets. Three cross‐cutting themes are emphasized, that is, yield potential, multiple stress tolerance and end‐user traits such as cooking time and canning quality. This study presents a review of the progress made in addressing major production constraints of common bean, hinging on the achievements of the Tropical Legumes (TL) project http://tropicallegumes.icrisat.org/ supported by the Bill and Melinda Gates Foundation (BMGF) being conducted by CIAT and the national bean programmes of Ethiopia, Tanzania and Uganda. The study also includes research conducted by other institutions on subjects relevant to the TL project. Three breeding pipelines are guiding the products being developed under the TL project. They include (i) bush and climbing bean breeding lines bred for drought tolerance, high mineral content, low P or N tolerance, (ii) bush and climbing bean breeding lines with heat and/or drought tolerance, and (iii) bush and climbing bean breeding lines for insect pest and disease resistance. Constraints to bean production are addressed through conducting strategic research to understand and utilize the available genetic diversity of the common bean (prebreeding), elucidate and exploit the biological basis of the bean plant for productivity gains, understand and utilize knowledge of the genetics and physiology to inform breeding strategies, and use this knowledge to develop superior client‐demanded varieties adaptable to environments in target regions.

## 3 PHENOTYPING PLATFORMS FOR KEY TRAITS

The need to validate and adapt screening protocols for prioritized traits, that is, BSM resistance, bruchid resistance, cooking time, canning quality, Fe and Zn grain content was raised by breeders in PABRA. Under the Tropical Legumes project, these protocols have been validated and are being used.

## 4 UNDERSTANDING AND UTILIZING GENETIC DIVERSITY FOR COMMON BEAN IMPROVEMENT

CIAT holds in trust the international *Phaseolus* genebank at its headquarters in Colombia, South America, and also maintains continuously increasing active collections of bean germplasm at the bean programme headquarters in Colombia and its Africa regional offices in Uganda (4,000 accessions currently) and Malawi (3,000 accessions currently). This huge diversity is tapped by plant breeders all over the world to continually improve specific traits. Four *Phaseolus* sister species, *P. coccinues*,* P. acutifolius*,* P. dumosus* and *P. costaricensis,* have been exploited to access unique genes for local selection (Beebe et al., 2013). *P. dumosus* and *P. coccineus* and *P. costaricensis* are native to competitive environments like that of wild common bean, but typically in somewhat more humid ecologies and are of interest as sources of resistance to diseases associated with humid environments (Singh, 2001). Tepary bean (*Phaseolus acutifolius*) evolved in the semi‐arid to arid environment where light is abundant and competition less intense, but moisture is severely limiting. Tepary bean also presents resistance to common bacterial blight (*Xanthomonas axonopodis), leaf miner (Empoasca kraemeri)* and bruchids (*Acanthoscelides obtectus)* (Singh, 1992).

## 5 EXPLOITING THE BIOLOGICAL BASIS FOR PRODUCTIVITY GAINS

Understanding the physiology of key traits helps breeder to promote faster breeding progress, improve selection methodologies and inform phenotyping protocols. In addition, enhancing the basic understanding of the biology of beans in general, traits of interest, for example, mechanisms of drought tolerance, yield, and virus resistance informs the breeding process. A study by Aruajo and Teixeria (2008) showed that grain yield of different common bean cultivars was not intrinsically associated with vegetative vigour at flowering and that mechanisms during pod filling could strongly influence the final crop yield. The establishment of a profuse root system during pod setting, associated with the continuous N and P acquisition during early pod filling, seemed to be relevant for higher grain yields of common bean.

### 5.1 Genetics, physiology and breeding for drought tolerance in common bean

Water scarcity, abundance, variability, date of onset of rains and length of the growing seasons or their combinations have direct impact on bean productivity. Drought limits the productivity of 50% of the arable land prompting competition for water (Cattivelli et al., 2008; Rosegrant, Ringler, & Zhu, 2009). Drought tolerance improvement will likely benefit 3.8 million ha in the 2020s (Beebe et al., 2011). When the Tropical legume project was initiated, breeding for drought tolerance in common bean that had received only sporadic attention gained prominence and moved into the research agenda. Nurseries consisting of materials segregating for drought tolerance and fixed lines combining drought tolerance with other traits, such as high mineral (iron and zinc) content and low soil fertility tolerance, bruchids resistance and common bacterial blight (CBB) disease resistance were distributed to the participating African countries, Ethiopia, Kenya, Malawi, Tanzania, Uganda and Zimbabwe. Genetic analysis was applied to a number of national bean collections, and high‐quality maps were developed for several populations. Physiological studies, directed for understanding drought tolerance and yield processes per se, revealed the underlying mechanisms of drought resistance and suggested how these could be applied within the breeding programmes. Visual rooting depth, root length at soil depth of 60 to 75 cm and carbon isotope discrimination in grain were shown to be valuable traits as selection criteria in breeding for drought stress tolerance in common bean (Beebe et al., 2013; Polania, Rao, Mejía, Beebe, & Cajiao, 2012). Remobilization of photosynthates from stems to pods and from pod walls to grain (Rao et al., 2013), pod partitioning index (PPI), harvest index (HI) and pod harvest index (PHI) (Polania et al., 2016) and basal root whorl number (BRWN) (Lynch, 2011) have also been identified as some of the physiological mechanisms governing drought tolerance. Breeders select for high yield potential under drought and irrigated conditions and also consider secondary traits such as Pod Harvest Index (PHI) in the selection index. Several drought QTL have been identified by CIAT Scientists (Blair et al., 2010; Diaz et al., in preparation), and at other institutes (Mukeshimana, Butare, Cregan, Blair, & Kelly, 2014; Trapp, Urrea, Cregan, & Miklas, 2015). Candidate QTL linked to PHI are being validated through additional phenotyping (Beebe et al., 2011). GWAS analysis of an 8‐parental MAGIC population revealed yield QTL on three chromosomes (Izquierdo et al., in preparation) which are in the validation process. However, other factors such as soil factors and poor soil fertility limit the expression of drought tolerance as they do not permit adequate plant development for crops to sustain additional physiological stress imposed by drought (Beebe et al., 2013, 2016).

### 5.2 Genetics, physiology and breeding for heat tolerance

Common bean is adapted to relatively cool climatic conditions, and temperatures of >30°C during the day or >20°C at night result in yield reduction (Porch, 2006). High temperatures were shown to aggravate the stress imposed by drought, and combinations of stress tolerance would be necessary in the near future (Beebe et al., 2016). Heat stress manifests in decline of photosynthetic leaf area, death of flowers, flower abortion, shortening of grain‐filling period, reduced pollen viability and seed weight, impaired development of yield components including ovaries (Hatfield et al., 2011) resulting in few seeds and decline in grain yield potential. Heat tolerance indices, geometric mean (GM) and stress tolerance index (STI) (Porch, 2006), extent of abscission of reproductive organs (Rainey & Griffith, 2005), chlorophyll a fluorescence (Stefanov, Petkova, & Denev, 2011) and pollen viability (Roman‐Aviles & Beaver, 2003) were found to be effective stress indicators of heat tolerance. While 20°C night temperature is normally considered to be a limitation for common bean, the breeding lines combining common bean with *P. coccineus* and *P. acutifolius* presented an excellent pollen formation and a good pod set at 22°C night temperatures and some pod set is maintained at 25°C nights (Roman‐Aviles and Beaver (2003). Heat‐tolerant bush (CIAT, 2016) and climbing bean lines (Blair, Iriarte, & Beebe, 2006) have been developed. Introgression of heat tolerance from *P. acutifolius* in backgrounds of more acceptable seed types and evaluating newly developed lines confirmed that *P. acutifolius* is an important and useful genetic resource for improving heat tolerance in common bean (Beebe et al., 2016; Polania et al., 2017).

### 5.3 Genetics, physiology and breeding for low soil fertility tolerance

Considerable genetic variability has been detected from field evaluations, and genotypes with specific single or multiple edaphic stress tolerance (low N, low P and soil acidity with the associated Al and/or manganese (Mn) toxicities) have been identified (Lunze et al., 2012). Long‐term research using common bean has contributed to defining root phenes and their role in enhanced soil exploration and P acquisition (Lynch, 2011). The genetics of N fixation and low P tolerance was evaluated by Diaz et al. (2017). Traits such as greater BRWN (Lynch, 2011), percentage of nitrogen derived from atmosphere (*%Ndfa*) (Mehdi, 2015; Rao, Miles, & Beebe, 2016), biological nitrification inhibition (BNI) (Subbarao, Yoshihashi, & Worthington, 2015), receptor kinases, transmembrane transporters, and transcription factors (Kamfwa, Zhao, Kelly, & Cichy, 2017) have been employed in selecting for tolerance to specific mineral deficiencies. Lines developed by CIAT for combined drought and low soil fertility tolerance are being evaluated in Ethiopia, Uganda and Tanzania. Some of these lines have been found to be resistant to Pythium and Fusarium root rot (Beebe et al., 2011).

## 6 GENETICS AND BREEDING FOR RESISTANCE TO MAJOR FIELD AND POSTHARVEST INSECT PESTS

Although a multitude of insect pests attack beans, bean stem maggots (BSM) (*Ophiomyia* spp, Diptera, Agromyzidae), white flies (*Bemisia tabaci*) that transmit bean golden mosaic virus disease (BGMV) and aphids (*Aphis fabae* and *Aphis craccivora*) that transmit bean common mosaic virus (BCMV) and its necrotic strain bean common mosaic necrosis virus (BCMV) and flower/pollen beetles (*Mylabris* spp and *Coryn*a spp) have been targeted in breeding programmes as they are considered most important field pests. Bean bruchids (*Zabrotes subfasciatus* and *Acanthoscelides obtectus*) are the major storage pest for the common bean.

Bean stem maggot (BSM) is generally regarded as the principal insect pest of beans throughout Africa causing up to 50%–100% yield losses especially when seedlings are attacked (Songa, 1999). Damage is more severe in infertile soils (Ochilo, 2013; Ampofo et al., 1998) and late‐sown crops (Ojwang, Melis, Githiri, & Songa, 2010). BSM infestation is aggravated by drought (Ojwang et al., 2010) and also occurs in association with bean root rots (Ochilo, 2013). The use of host plant resistance against BSM is supposed to be more effective in the management of BSM (Abate, 1990; Murenju, 2015) though not absolute (Belmain, Haggar, Holt, & Stevenson, 2013). The black bean aphid (*A. fabae*) is the main aphid pest of beans and causes direct damage wherever the crop is grown in Africa. The cowpea aphid (*A. craccivora*) may also colonize bean plants especially in low altitudes. Little has been carried out to breed for resistance to this pest with more attention being given to the virus disease (BCMV/BCMNV) that is transmitted by this pest. White flies have a wide host range that includes many crops and weeds. Low levels of whiteflies do not cause much damage and do not warrant control interventions. The adults may transmit the cowpea mild mottle virus and bean golden mosaic virus (BGMV) (Costa, 1965). BGMV has not been reported as important in Africa. However, with the changing climate, the increase in whiteflies populations is feared to have an impact on bean productions as a pest and as a virus vector. Identification of sources of resistance to the bean flower/pollen beetles (*Mylabris* spp and *Coryna* spp) is underway in Uganda. Bean bruchids are widely distributed in Africa. The larvae of both weevils can stay undetected in the seed until the adult emerges. Antibiosis expressed as adverse effects of seed protein arcelin in extending the time of adult emergence, growth and life cycle of these insects (Velten, Rott, Conde‐Petit, Cardona, & Dorn, 2008) in wild bean accessions has been exploited in developing bruchid‐resistant common bean germplasm. Bean genotypes with arcelin based resistance have been developed (Cardona, 2004; Beneke, 2010) and markers tagging this resistance also developed.

## 7 GENETICS AND BREEDING FOR RESISTANCE TO KEY DISEASES IN AFRICA

Diseases are the second most important constraints to bean production, after abiotic factors in Africa causing up to 80%–100% yield loss (Wortmann et al., 1998). Major success has been in breeding for resistance to angular leaf spot, anthracnose, and common bacterial blight, bean root rot and bean common mosaic virus. Although resistance to angular leaf spot (ALS) (*Psuedocercospora griseola*) is mostly a monogenic trait, the pathogen is highly variable with many different races (Mahuku, Henriquez, Munoz, & Buruchara, 2002). Three ALS resistance genes are mapped and named following the guidelines for gene nomenclature proposed by the Bean Improvement Cooperative (BIC) Genetic Committee: Phg‐1 (AND 277) on chromosome Pv01 (Carvalho et al., 1998; Gonçalves‐Vidigal et al., 2011), Phg‐2 (Mexico 54) on Pv08 (Sartorato, Nietsche, Barros, & Moreira, 2000) and Phg‐3 (Ouro Negro) on Pv04 (Corrêa et al., 2001; Gonçalves‐Vidigal et al., 2013). However, in addition to these genes, unnamed major resistance loci were reported in different resistance sources used by common bean breeding programmes in Uganda, Colombia and Brazil. The major QTL ALS4.1GS, UD on Pv04, present in G5686, and the ALS10.1DG, UC on Pv10, identified in both G5686 and CAL143 (Keller et al., 2015), were officially named as Phg4 and Phg‐5 (Souza, Gonçalves‐Vidigal, ABREU, & Pastor‐Corrales, 2015). Gene pyramiding has been suggested to provide resistance to a wide range of the ALS pathotypes (Miklas, Kelly, Beebe, & Blair, 2006; CIAT, 2007). Bean anthracnose is a highly variable pathogen, new pathotypes reportedly keep emerging time after time (Leaky and Simbwa‐Bunya, 1972; Nkalubo, 2006; Pastor‐Corrales & Tu, 1989). Resistance to this pathogen is conditioned by nine independent resistances (*Co1‐Co‐10*). Information on pathogenic variability present in production areas is essential in designing effective gene pyramids in addition to continued evaluation of resistance sources as the genes differ in their effectiveness in controlling variable races. The genotype G2333 which possesses *Co‐4* ^*2*^, *Co‐5* and *Co‐7* resistance genes (Young, Melotto, Nodari, & Kelly, 1998) has been utilized routinely in introgressing Anthracnose resistance. Gene pyramiding is suggested to provide efficient long‐term control of bean anthracnose (Balardin & Kelly, 1998). Common bacterial blight (CBB) resistance is conditioned by polygenic genes, and 24 QTL have been identified across all 11 linkage groups/chromosomes making breeding for genetic resistance complex (Singh & Schwartz, 2010). Using traditional breeding approaches, bean cultivars and lines with improved CBB resistance were developed by combining resistance sources from the primary and secondary gene pools with *P. acutifolius*) as the major source of resistance (Singh, Munoz, & Teran, 2001) and resistant lines developed. New sources of resistance have been identified (Alladassi et al., 2017). With MAS, the accumulation of QTL from diverse sources may now be attempted to attain higher levels of CBB resistance in new bean cultivars. Resistance to Pythium root rot has been demonstrated to be simply inherited and conditioned by single dominant genes (Otysula, 2003). Resistance to Fusarium root rot (*Fusarium solani* f.sp. *phaseoli*) was demonstrated to be quantitatively inherited (Mukankusi et al., 2011). QTL related to FRR resistance and root/shoot biomass were identified in RIL populations of MLB‐49‐89A (Weijia et al., in press) and Puebla 152 (Nakedde et al., 2016). Bean common mosaic virus (BCMV) and bean common mosaic necrosis virus (BCMNV) are the most widespread and important viral diseases affecting production of common beans in Africa (Spence & Walkey, 1995) causing up to 80% yield loss (Morales, 2003; Wortmann, 1998). A number of BCMV and BCMNV resistance genes have been identified and tagged. They include the single dominant *I* and the recessive *bc*‐*u, bc‐1, bc‐1* ^*2*^, *bc‐2, bc‐2* ^*2*^ and *bc‐3 g*enes (Drijfhout, 1978; Miklas & Kelly, 2002). The dominant *I* gene inhibits all known strains of the BCMV (Drijfhout, 1978). However, due to occurrence of BCMNV (a fact with consequences that were unknown when the “*I”* gene was introduced in bean lines in Africa) germplasm containing the I gene (introduced or developed in the region) faced an unanticipated problem. When the “*I”* gene containing material is invaded by the BCMNV strains, the “*I”* gene responds by producing excess phaseolin in the vascular system of the inoculated leaf and in the plant apex. This results in the death of the apex (a condition known as systemic top necrosis or black root) and discoloration of the vascular tissue due to downward movement of phaseolin, and eventually death of the plant (Kelly, 1997). This limits their usefulness of germplasm with the dominant I gene in the presence of the BCMNV strains. Protection of the “*I”* gene by combining it with race‐specific resistance recessive genes (typically *bc‐3 or bc‐2* ^*2*^) and introgressing this resistance into key materials that neither have “*I”* gene nor any other type of resistance against BCMV or BCMNV have been used as the most suitable strategies to provide stable and broad‐based resistance.

### 7.1 Biofortification in common bean

Biofortification research was initiated following justification of the prevalence of high levels of undernutrition due to nutrient deficiencies including iron deficiency anaemia (Petry et al., 2007; Mulambu et al., 2017; Blair et al., 2013). Grain mineral levels ranging from 30 to 110 ppm for iron and 25–60 ppm for zinc have been found from screening of bean germplasm accessions from the global gene bank and local collections from ten African countries (Pfeiffer & McClafferty, 2007; Mukamuhirwa, Tusiime, & Mukankusi, 2015; Mulambu et al., 2017). The highest concentrations were often found in progenitors or wild relatives of common bean (Beebe et al., 2000; Islam, Basford, Jara, Redden, & Beebe, 2002). Substantial positive associations (60%–80%) were discovered between iron and zinc levels, which provided an opportunity for raising levels of both micronutrients simultaneously (Pfeiffer & McClafferty, 2007). Early product development involved identifying parental genotypes for use in crosses and understanding the genetics of the trait (Mulambu et al., 2017). High‐iron genotypes were used to conduct crosses (including double‐crosses with two or three high‐iron parents) to combine the high‐mineral trait with acceptable grain types and agronomic characteristics (Beebe et al., 2000) including wide crosses with *P. dumosus* and *P. acutifolius* (Beebe, 2012a; Beebe et al., 2012b). Genotype‐by‐environment (GxE) tests were conducted to verify that mineral accumulation was stable across sites and generations (Blair et al., 2010; Mukamuhirwa et al., 2015). The small‐seeded Mesoamerican bush bean lines emerging from the breeding programme in Colombia have 80% higher iron and drought resistance that was equal to or superior to the tolerant check (Beebe et al., 2016). The improvement of mineral levels in climbing bean materials has also been most successful and had an added advantage of increased productivity per unit area (Beebe et al., 2016). Further improvements can be achieved because nutrient content was shown to be positively correlated with high yield potential and genotype x environment effects were small (Bationo, Waswa, Kihara, & Kimetu, 2007).

### 7.2 Marker‐assisted selection in common bean

Most progress with marker‐assisted selection (MAS) in common bean breeding has been with disease resistance. Through the TL project, communication was established between CIAT and USDA to access sequence data to identify SNP markers. Under the Generation Challenge Program (GCP), ~1,500 SNPs available through the BeanCAP project were converted to the KasPAR system at a genotyping outsourcing service provider. SNP markers for major disease resistance genes (for BCMNV, BGMV, CBB, bruchids, ALS) were developed, and markers of other classes (SCARS, SSRs) have been converted to a SNP platform for ready to use in gel‐free systems for in‐house genotyping or through the genotyping service provider (Table 2). Currently, two SNP genotyping providers offer services for single‐locus genotyping in common bean, LGC (UK) and Intertek Group Plc (Sweden). The latter has recently been added through the high‐throughput project for genotyping (HTPG), to offer cost‐effective genotyping service for breeders mainly in the CGIAR system. In addition, sequencing platforms have been developed. An evaluation of available whole‐genome sequence data sets was analysed, revealing intergene pool and interspecific introgressions in breeding material (Soler et al., 2017, submitted), and several publications show data on genotyping by sequencing (GBS) (Ariani, Teran, & Gepts, 2016; Ferreira, Murube, & Campa, 2017; Moghaddam et al., 2016; Schröder et al., 2016) mostly using GBS based on the protocol from Elshire et al., 2011. A GBS‐related SNP platform, Integrated Genotyping Service and Support (IGSS), has been set up at ILRI‐BECA to respond faster to African plant breeders needs. Sequencing data are used to select SNPs for marker design, or for genetic studies of whole populations. In genomic selection (GS), these high‐density genotyping methods may also be used directly in breeding.

**Table 2:** List of new SNP markers developed for use in common bean breeding programmes

Marker ID	Intertek name	LGC name	Source Genotype	References	Chromosome	Trait	Gene/QTL
ALS_Phg2_08_GT_61901182	snpPV0071		G10474	CIAT	8	ALS	phg‐2
ALSChr04_GC_43800347	snpPV0032	ALSChr04_GC_43800347	G5686	Lobaton et al., accepted for publication	4	ALS	phg‐4
ALSChr08_CT_57798588	snpPV0033	ALSChr08_CT_57798588	G10474	”	8	ALS	phg‐2
MAS_ALS10a	snpPV0025	MAS_ALS10a	G10474	”	8	ALS	phg‐2
MAS_ALS10c	snpPV0027	MAS_ALS10c	G5686	”	10	ALS	phg‐5
MAS_ALS4b	snpPV0029	MAS_ALS4b	G5686	”	4	ALS	phg‐4
ANT_Co‐1_ss715646578	snpPV0048		G122, AFR298, Montcalm	Zuiderveen et al. (2016)	1	ANT	Co‐1
ANT_Co‐4_08_CG_2329860	snpPV0069		G2333	Oblessuc (2010); Burt et al., 2015.	Chr08	ANT	Co‐4
ANT_Co‐u_ss715648452	snpPV0045	ANT_Co‐u_ss715648452	Montcalm	Zuiderveen et al. (2016)	2	ANT	Co‐u
bc‐3a	snpPV0001	bc‐3a	SCR42	Hart and Griffiths (2015), Naderpour et al. (2012)	Chr06	BCMV	bc‐3
Bc‐3b	snpPV0002	bc‐3b	SCR42	”	Chr06	BCMV	bc‐3
MAS_BC‐3B1	snpPV0003	MAS_BC‐3B1	BRB 191, MAZ42, DABA 60, MAZ 34	”	Chr06	BCMV	bc‐3
BCMV_I_00453_M1	snpPV0004	BCMV_I_00453_M1	Montcalm	Bello et al. (2014)	2	BCMV	I
BRU_00261	snpPV0007		RAZ124	CIAT	4	Bruchid	APA
BRU_00262	snpPV0008		MAZ26, MAZ13, MAZ21, MAZ32	CIAT	4	Bruchid	APA
BRU_IntRegAPA3	snpPV0006	IntRegAPA3	MAZ42	Blair et al. (2010)	Chr04	Bruchid	APA
CBB_SAP6_801	snpPV0038	SAP6_801	VAX1,3,4,5,6	Lobaton et al., accepted for publication	10	CBB	
CBB_SU91_g91004686	snpPV0039	SU91_g91004686	VAX3,4,5,6,	”	8	CBB	
lpa_chr01_42595000_C_T	snpPV0067		lpa127	CIAT	1	lpa	Pvmrp1

ALS, angular leaf spot; ANT, anthracnose; CBB, common bacterial blight; BCMV, bean common mosaic virus; lpa, low phytic acid.

The *bc‐3* gene is the only allele with a known mechanism of resistance to bean common mosaic virus (BCMV) disease and its necrotic strain, bean common mosaic necrosis virus (BCMNV). The bc‐3 gene is also identified as the *eIF4E* allele carrying a mutated eukaryotic translation initiation factor gene (Naderpour et al. 2010). To date, a CAPS and SNP marker based on the eIF4E gene have been developed for utilization using the Intertek and LGC platforms (Table 2). Similarly, SNP markers have been developed for the SCAR marker (SW13_690_) by Bello et al. (2014) and validated by Melotto, Afanador, and Kelly (1996) and others (CIAT, 2003) for MAS. The SW13_690_ marker is linked to the dominant *I* gene that confers resistances to BCMV but results in a necrotic reaction in the presence of BCMNV when the bc‐3 gene is absent. The dominant *I* gene and recessive *bc‐3* gene have been transferred from small‐seeded Mesoamerican bean cultivars to the large‐seeded Nueva Granada types at CIAT. Using MAS techniques, the most effective bean anthracnose resistance gene Co‐4^2^ was transferred into adapted pinto bean lines in less than 18 months (Miklas & Kelly, 2002). GWAS for Anthracnose resistance in the ADP panel revealed three groups of race‐specific QTL and SNPs (Zuiderveen, Padder, Kamfwa, Song, & Kelly, 2016). A SNP marker linked to Co‐4^2^ gene has been developed and deployed for utilization in the Intertek platform (Table 2). SCAR markers (SCAreoli_1000_, SAS13_950_, SH181_1100_, SBB14aa_1150/1050_, SAB3_400_, OPAZ20_940_ and SAB12_350_) linked to the majority of the major Co‐genes that confer resistance to anthracnose have been reported widely and provide an opportunity to enhance disease resistance through MAS. A total of 12 markers were identified to be linked in coupling to the Pythium root rot resistance gene (Buruchara & Kimani, 1999). Three RAPD primers were successfully converted to SCAR markers at 1.5 cM (PYAA19_800_), 4.0 cM (PYBA08_350_) and 6.0 cM (PYY20_1200_) from the resistance gene. The PYAA19_800_ SCAR marker was validated and successfully used in selection for Pythium root rot resistance (Mahuku, Buruchara, Navia, & Otsyula, 2007). Ongom, Nkalubo, Gibson, Mukankusi, and Rubaihayo (2012) confirmed that the PYAA19800 SCAR marker was strongly associated with *Pythium ultimum* resistance and not linked to Fusarium root rot resistance. Using simple sequence repeats (SSR) markers, Kamfwa, Mwala, Okori, Gibson, and Mukankusi (2013) found a significant major QTL for resistance to Fusarium root rot in the resistant line MLB‐49‐89A. The study also found that the two markers PVBR87 and PVBR109 spanning the QTL are found on B3 of the common bean core map close to the region where resistance to root rots, anthracnose, common bacterial blight and bacterial brown spot have been previously mapped (Kamfwa et al., 2013). RAPD markers that are tightly linked to angular leaf spot resistance genes were identified and some successfully converted to SCAR markers (CIAT, 2003; Namayanja et al., 2006). The protocol for their use in marker‐assisted selection breeding was also developed (Mahuku, Jara, Cajiao, & Beebe, 2003). The utility of one of the markers, SCAR‐OPE_709_, has been demonstrated in segregating populations of different backgrounds (Ddamulira et al., 2015). A SCAR marker (PF9 260_G1_) was identified in G10474 and G10909, and SNP markers linked to resistance in G10474, G5686, AND277 and MAB lines have been generated at LGC and Intertek for utilization by breeders. Three SCAR Markers BC420, SU91 and SAP6 linked to three major QTLs are being used for MAS of CBB resistance in East Africa. New SNP markers were developed for CBB to replace SCARs (http://www.integratedbreeding.net). A bruchid resistance evaluation nursery trial at CIAT HQ showed good correlation of the APA marker with resistance. APA_SNP_Chr04_44239098_G_A and APA_SNP_Chr04_44350220_A_G. markers are being utilized to select for resistance to *Zabrotes* sp in three populations (Awash‐1 × RAZ11; Awash‐1 × RAZ42; Awash‐1 × RAZ120) originating from Ethiopia using a real‐time PCR platform.

### 7.3 Genomic selection

Genomic selection (GS) is a recently developed breeding method based on evaluating a training population phenotypically and genotypically to develop a phenotype prediction model. This model is then used to predict phenotype or phenotypic potential in early generation materials based on genotypic data. This method is promising to increase selection precision, to accelerate breeding cycle times and to predict performance in distant target areas. Based on the 8‐parental MAGIC population data, prediction precisions of up to 0.7 were observed for high heritability traits like 100 Seed Weight. For more difficult traits like Yield, predictions were lower ~0.3. Prediction of 2014 phenotypic data with a 2013 data model had a precision of ~0.5 for 100SDW (correlation between years: 0.67) for YD ~0.2 (correlation 0.35). Results show some promise that more models and populations will be evaluated comparing more sites and seasons using breeding panels as training populations.

### 7.4 Breeding data management

Identifying strategies for the sustainable intensification of smallholder farming systems requires measurements of key crop performance traits under local field conditions. Tools needed to make these measurements, process the data and extract useful information have been out of reach of most researchers, extension agents and farmers in Africa for a long time. Accurate cultivar performance data over a period of time are essential for making predictions for the future. The breeding management system (https://www.integratedbreeding.net/) is a suite of interconnected software designed to help breeders manage day‐to‐day activities through all phases of their breeding programmes: from straightforward phenotyping to complex genotyping, providing necessary tools to conduct modern breeding in one comprehensive package, local database for a breeder to track. Under the Tropical legume III project, three countries Ethiopia, Uganda and Tanzania are uploading bean breeding data into the BMS. A server has been installed at CIAT HQ, and the first field books have been uploaded with the plan to follow some breeding generations with BMS in 2016 in parallel with the current data management system. The use of electronic data collection gadgets is also being streamlined across these countries to help speed up the process of data collection and reduce errors. Servers of the BMS have also been installed at NaCRRI and EIAR.

## 8 VARIETY DEVELOPMENT AND RELEASE

The CIAT bean programme is promoting principles that drive success in demand‐led breeding that include (i) target‐driven breeding approach; (ii) demand‐led variety development strategy; and (iii) performance indicators to measure progress towards the adoption and widespread use of new plant varieties (Persley & Anthony, 2017). Product profiling is one of the best practices under a target‐driven approach to variety development. This includes defining the type of product being developed and the market. In addition, factors that would affect the development of that product are also outlined and a breeding scheme designed with a timeline (Tropical legume III report, 2017). Product profiles for seven grain market classes that include large white, large red, small white, small red, large red speckled (sugar bean), large red mottled (calima type) and medium‐to‐large yellow beans were developed across the three countries, Ethiopia, Tanzania and Uganda. An example of a product profile for small white beans for Ethiopia bean programme is shown in Table 3. Under the TL project, a total of 71 market‐demanded varieties with on‐farm yield advantage of 10%–40% over the commercial varieties and additional traits of resistance to key pests and diseases and/or high grain Fe and Zn content were released in six countries, Ethiopia, Kenya, Malawi, Tanzania, Uganda and Zimbabwe over a 10‐year period (Table 4).

**Table 3:** Product profile for Small white bean for export market with drought and disease tolerance in Ethiopia

Consideration	Description
Target agroecology:	Lowland to mid‐altitude (1,000–1,900 masl) + rainfall (min. 500 mm/year)
Producer	Smallholder farmer and commercial producers, 119,000 Ha production, 921,000 households, 95% total white bean area, 38% total bean area
Customer:	Export market (>95%)
Yield potential:	≥standard check (Awash2)
Maturity:	Early (70–90 DM)
Abiotic stresses:	Drought tolerance (intermittent + terminal)
Diseases:	CBB (≤6), rust (≤6), HB (≤6), anthracnose (≤6)
Seed size:	20–25 g/100 seeds
Seed colour:	White
Seed shape:	Round or oval (must be distinct)
Quality:	Hydration coefficient ≥1.8; % washed drained wgt ≥60%; canning liquid must be clear not turbid; splitting ≥7; clumping ≥4; no bleaching after canning
Future traits:	Bruchid resistance (100%)

**Table 4:** Varieties released (2008–2017) in six countries supported by the Tropical legume project

Official name	Year of release	Maintainer	Country
1. Dursitu	2008	EIAR	Ethiopia
2. Kufanzik	2008	EIAR	Ethiopia
3. Deme	2008	EIAR	Ethiopia
4. Hawassa Dume	2008	EIAR	Ethiopia
5. Batu	2008	EIAR	Ethiopia
6. GLP‐2	2011	EIAR	Ethiopia
7. ECAB‐0056	2012	EIAR	Ethiopia
8. ECAB0060	2013	EIAR	Ethiopia
9. ACC4	2013	EIAR	Ethiopia
10. RXR10	2013	EIAR	Ethiopia
11. K132	2013	EIAR	Ethiopia
12. ECAB0203	2013	EIAR	Ethiopia
13. ECAB0247	2013	EIAR	Ethiopia
14. KATB9	2014	EIAR	Ethiopia
15. KATB1	2014	EIAR	Ethiopia
16. NAVY 87	2014	EIAR	Ethiopia
17. SER 119	2016	EIAR	Ethiopia
18. SER 125	2016	EIAR	Ethiopia
19. Bifort small seeded – 15	2017	EIAR	Ethiopia
20. DAB 96	2017	EIAR	Ethiopia
21. F10 B. sel new Bilfa 58	2017	EIAR	Ethiopia
22. Biofort large seeded – 5	2017	EIAR	Ethiopia
23. BZ‐2	2017	EIAR	Ethiopia
24. DAB277	2017	EIAR	Ethiopia
25. MR14 152‐43‐2P	2017	EIAR	Ethiopia
26. DAB489	2017	EIAR	Ethiopia
27. SCR‐26	2017	EIAR	Ethiopia
28. NABE 17	2012	NARO	Uganda
29. NABE 18	2012	NARO	Uganda
30. NABE 19	2012	NARO	Uganda
31. NABE 20	2012	NARO	Uganda
32. NABE 21	2012	NARO	Uganda
33. NABE 22	2012	NARO	Uganda
34. NABE 23	2012	NARO	Uganda
35. NABE 26	2012	NARO	Uganda
36. NABE 27C	2012	NARO	Uganda
37. NABE 28C	2012	NARO	Uganda
38. NABE 29C	2012	NARO	Uganda
39. NAROBEAN 1	2016	NARO	Uganda
40. NAROBEAN 2	2016	NARO	Uganda
41. NAROBEAN 3	2016	NARO	Uganda
42. NAROBEAN 4	2016	NARO	Uganda
43. NAROBEAN 5	2016	NARO	Uganda
44. Njano‐Uyole	2008	ARI Uyole‐Mbeya	Tanzania
45. Calima Uyole	2011	ARI Uyole‐Mbeya	Tanzania
46. Fibea	2012	ARI Uyole‐Mbeya	Tanzania
47. Pasi	2012	ARI Uyole‐Mbeya	Tanzania
48. Rosenda	2012	ARI Uyole‐Mbeya	Tanzania
49. Uyole Nyeupe	2016	ARI Uyole‐Mbeya	Tanzania
50. Uyole 16	2016	ARI Uyole‐Mbeya	Tanzania
51. Uyole Nyeupe	2016	ARI Uyole‐Mbeya	Tanzania
52. KATB9	2017	ARI‐Selia, Arusha	Tanzania
53. KATB1	2017	ARI‐Selia, Arusha	Tanzania
54. MAC 44	2017	ARI‐Selia, Arusha	Tanzania
55. RWV1129	2017	ARI‐Selia, Arusha	Tanzania
56. SWP‐09	2017	ARI‐Selia, Arusha	Tanzania
57. SWP‐11	2017	ARI‐Selia, Arusha	Tanzania
58. SWP‐12	2017	ARI‐Selia, Arusha	Tanzania
59. KAT‐SR 01	2012	KALRO‐Katumani	Kenya
60. KAT‐RM‐001	2013	KALRO‐Katumani	Kenya
61. VTTT 924/4‐4	2012	DAR‐Chitedze	Malawi
62. SER 124	2013	DAR‐Chitedze	Malawi
63. VTTT 925/9‐1‐2	2013	DAR‐Chitedze	Malawi
64. SER 83	2013	DAR‐Chitedze	Malawi
65. BF 13607‐9	2013	DAR‐Chitedze	Malawi
66. CIM 9314‐17	2012	CBI‐DRSS	Zimbabwe
67. SUG 131	2012	CBI‐DRSS	Zimbabwe
68. Gloria (PC652‐SS3)	2012	CBI‐DRSS	Zimbabwe
69. NUA 45	2012	CBI‐DRSS	Zimbabwe
70. MG 38	2013	CBI‐DRSS	Zimbabwe
71. VTTT 925/9/1/2	2013	CBI‐DRSS	Zimbabwe

EIAR, Ethiopia Institute of Agricultural Research; NARO, National Agricultural Research Organisation; ARI, Agricultural Research Institute; KALRO, Kenya Agricultural and Livestock Research Organisation; DAR, Department of Agricultural Research; CBI‐DRSS, Crop Breeding Institute‐Department of Research and Specialist Services.

## 9 GENDER‐RESPONSIVE BEAN BREEDING

The primary goal of bean breeding is to increase production in highly heterogeneous environments. Under the PABRA framework, effort has been made to integrate gender in the breeding process especially in participatory variety selection. Farmer participatory variety selection (PVS) is a step included in the later stages of the bean breeding process to ensure acceptability and eventual adoption (Gyawali, Sunwar, & Subedi, 2007) of developed varieties. It entails farmers and other stakeholders evaluating large number of varieties to provide feedback to breeders on their own preferences in the process of variety selection sequence. PVS has been institutionalized within CIAT and NAREs and has become a norm rather than an exception. A review of findings from strategic survey of PVS methods for three countries of East Africa, Uganda, Kenya and Tanzania, highlighted the importance of integrating choice experiment approaches in understanding trait preferences differentiated by sex (men vs women) or generation (old vs youth farmers) and household income status (rich vs poor). Empirical evidence consistently showed that farmers use a diverse range of criteria to select bean varieties that meet their priorities. From the farmer participatory variety selection results from Kenya and Uganda activities and surveys conducted in Kenya in 2012 and Uganda (2012–2013), key traits generally preferred by men and women bean growers across various production context include yield potential, taste and marketability. However, with the exception of taste, the importance attached to each trait may vary across the social groups (men vs women) depending on the gender roles within the value chain. For example, empirical evidence shows that criteria such as texture of bean leaves, keeping quality and cooking time are more important to women than men (Katungi et al., 2011). In Kenya, men are more likely than women to reject varieties with climbing habits as they interfere with the growth of their maize crop (Katungi et al., 2011). Consequently, breeding has maintained its focus on achieving key acceptable traits (i.e., yield, resistance, marketability and taste) while minimizing those that will lead to rejection.

## 10 CONCLUSIONS

The need to continuously develop suitable varieties that match requirements for a changing climate and changing market demands remains. The good genetics developed by CIAT should be complimented by sound agronomic practices. This calls for relevant and low‐cost soil and water management practices which can enhance adaptation of the new multiple stress‐tolerant varieties to low soil fertility and drought. The technology requires gender relevance and acceptance given that women are important for a crop like beans and are crucial in influencing change. However, there is a need to tackle several challenges which can compromise product delivery especially in the areas of limitations of social science capacity and limited research. Unclear and explicit strategies to deal with climate change effects may also hinder progress. A system approach that is cognizant of the fact that African farmers grow a multitude of crops which also buffers them from climate shocks should be adopted. Breeding efforts could possibly address constraints of the bean crop when grown in a system, most commonly in combination with cereal crops, agroforestry systems and in a mixed farming where livestock rearing is part of the component. Nutrient use efficiency in climate constrained environments could possibly be an area of focus. One of the major factors affecting the vibrancy of breeding programmes in Africa is the lack adequate support to adequately develop and follow through breeding strategies that respond to the needs of the country guided by market, consumer and environmental surveys. African breeding programmes are characterized by intermittent funding and ever‐changing priorities that affect continuity of promising programmes. Breeding programmes require smooth and uninterrupted support to operate and continually provide the genetic gains and products that are sought after by the beneficiaries. Continued and frequent assessment of the breeding programmes to inform improvement plans are key in ensuring that they remain relevant and investment worthy. Adoption of novel and efficient (timely, accurate and cost‐effective) breeding tools (e.g., phenotyping, genotyping, image analysis, data management etc.) requires continuous capacity building of upcoming breeders and technical staff and goes hand in hand with easy accessibility of these tools and the much‐needed research infrastructure. Adoption of innovative systems to reduce the breeding process is key in meeting the demands that come from climate change and consumer dynamics. Much of genetic gain made at CIAT today is emerging from interspecific crosses with sister species of common bean. Efficient, effective and rapid system for exploiting this diversity, for identification of elite lines for varietal release, and for recycling breeding lines through the hybridization programme, is a requirement to meet the challenges of crop improvement. Climbing beans are an especially promising option. With far higher yields that can triple those of bush beans, climbing beans have a niche in mountainous regions of high population density and limited land availability. Social science research on gender impacts of climate change (such as women's land ownership, access to water and their specific requirements for bean varieties) should be prioritized and improving on capacity building for communities to get the best of the new beans. This includes looking at possibilities of implementing projects on drought insurance products as part of the mitigation strategies in SSA which was proposed long ago (Nieto et al. 2012). Institutionalization through inclusion of the demand‐led breeding principles in breeding activities of the NARS breeding programmes will see a growth of competitive bean varieties that demand sizeable shares in local, regional and international markets. Limited access to accurate and timely information on climate could hinder progress in breeding as it hinders pre‐emptive breeding. It also slows down the breeding process and in many cases could result in resource wastage if not well prioritized. This faces the possibility for funding agencies to change priorities. It should accelerate implementation of projects because funders can change priorities. Finally, partnerships are important to ensure that investments in bean research have more impact. One of the major reasons for the current successes in bean research in Africa has been due to the PABRA model. The PABRA model comprises partnerships between and among International Centre for Tropical Agriculture (CIAT), National Agricultural Research Systems (NARS), public‐ and private‐sector actors along the varied bean product value chains, and technology end users (Buruchara et al., 2011). The model promotes principles that include the enhancement of synergy and efficiency among partners, building of social capital, partnership and leveraging comparative advantage of partners, strengthening national ownership of programmes, inclusion of new and potential/common actors (seed companies, NGOs), building on NARS bean programmes and existing partner networks, linkages with other big initiatives (several seed companies and donor supported and shared responsibility among PABRA members).

## CONTRIBUTION OF AUTHORS

Clare Mukankusi, Reviewed and compiled contributions from co‐authors and reviewed progress of Tropical Legume project‐breeding component. Reviewed literature from other bean communities supporting findings of the Tropical Legume project. Provided progress of the bean breeding programme at CIAT‐Uganda under the Tropical Legume project; Bodo Raatz, Developed and provided the review of current SNP markers developed at CIAT that are available for the common bean community. Provided progress of the Andean bean breeding programme at CIAT's Headquarters in Colombia; Stanley Nkalubo, Provided information pertaining to common bean breeding progress of the Tropical Legume project in Uganda; Berhanu Fenta, Provided information pertaining to common bean breeding progress of the Tropical Legume project in Ethiopia; Papias Binagwa, Provided information pertaining to common bean breeding progress of the Tropical Legume project in Northern Tanzania under the TL project; Michael Kilango, Provided information pertaining to common bean breeding progress of the Tropical Legume project in Southern Tanzania; Magdalena Williams, Provided information pertaining to common bean breeding progress of the Tropical Legume project in western Tanzania; Katungi Enid, Reviewed progress of the social economics work in particular gender‐responsive participatory common bean variety selection conducted by the Tropical Legume project; Rowland Chirwa, Provided common bean breeding progress of the Tropical Legume project at CIAT Malawi and the National programmes of Malawi and Zimbabwe; Steve Beebe, Provided progress of the Mesoamerican common bean breeding programme at CIAT Headquarters in Colombia under Tropical Legume TL project.

## CONFLICT OF INTEREST STATEMENT

This paper is a review of the major outputs of the Tropical legume (TL) project Common bean breeding, genomics and genetics. It includes findings of the project but also highlights findings of other researchers on specific topics. As such, there is no conflict of interest.
